# First-rotation growth and stand structure dynamics of tree species in organic and conventional short-rotation agroforestry systems

**DOI:** 10.1016/j.heliyon.2018.e00645

**Published:** 2018-06-07

**Authors:** J.A. Huber, M. Matiu, K.-J. Hülsbergen

**Affiliations:** a Technische Universität München, Chair for Organic Agriculture and Agronomy, 85350 Freising, Germany; b Technische Universität München, Professorship Ecoclimatology, 85350 Freising, Germany

**Keywords:** Agriculture, Environmental science, Plant biology

## Abstract

Short-rotation agroforestry systems can potentially maintain agricultural production and promote conservation of soil and biodiversity, especially if grown organically. Hereby, species-specific stand growth determines woody biomass yield and influences management decisions like planting density and harvest requirements. Studies of longer-term growth dynamics in Southern Germany are scarce and none analyzed differences between conventional and organic systems. In this study, four tree species (black alder, black locust, poplar clone Max 3, and willow clone Inger) were planted in an alley-cropping configuration in Southern Germany, grown under organic and conventional systems, and monitored from 2009 to 2012. Growth was assessed with stem base diameter, height, aboveground woody biomass, sprouting, and survival. The tree species did not show a uniform ranking in biometric variables and biomass over time. Four-year mean annual biomass increment (MAI) ranged from 7 to 10 t ha^−1^ a^−1^, with poplar and locust having the highest growth rates. Willow had the lowest MAI, as it had a low diameter growth paired with a low wood density, but it developed the highest number of shoots because of increased sprouting in the last year. Size inequality and skewness of the dominant stems increased for all species throughout the years suggesting asymmetric competition. Size inequality as well as mortality was greatest for black locust. Furthermore this was the only species, which developed a right skewed SBD distribution and the highest diameter size range. Size inequality was smallest for poplar and willow, with no or only minimal mortality. Alder was inbetween. For black locust and alder no difference in growth traits between organic and conventional systems appeared after four years. Organic poplar and willow stands performed better than conventional ones after the second year, leaving unclear whether this can be attributed to management or site effect.

## 1 Introduction

Biomass from short rotation agroforestry systems (SRAFS), mostly planted as alley cropping configuration, and short rotation coppices (SRC) have garnered great interest as feedstock for renewable energy. By sequestering carbon and substituting fossil fuels, those systems help to mitigate climate change and reach the EU climate and renewable energy policy targets [1]. In addition, multiple positive environmental impacts are provided by implementing such systems, including biodiversity benefits and soil and water protection [2, 3].

To be commercially feasible as well as to enhance carbon storage and energy use efficiency, high yields have to be sustained. Hybrids of the poplar and willow genera were pronounced to be the most yielding species [4, 5, 6]. Also locust and alder were found to be well suited as short rotation crops, especially on less fertile sites, where they benefit from their ability to fix atmospheric nitrogen [7, 8, 9, 10]. Depending on species-specific growth patterns, ecophysiological mechanisms and interactions, the performance of individual species and clones varies during and between rotations according to site conditions (climate, soil properties, diseases, insects) and management (fertilization, irrigation, planting density, weed control, planting configuration). Therewith, in SRC a wide range of yield dynamics have been reported for poplar [11, 12, 13, 14, 15], willow [16, 17, 18, 19], but also within family variation for alder [20] and black locust [7]. Although some studies exist on the suitability of various tree species as short rotation crops in Germany [8, 10, 21, 22], only few were conducted in southern Germany on fertile [23, 24, 25] and marginal land [25, 26]. Furthermore, only few studies dealt with the growth performance in SRAFS with an alley configuration, where edge effects highly influence total woody biomass yields [22]. Even less studies were performed at organic SRAFS [25, 27], and to the authors' best knowledge none that compares organic and conventional systems.

Besides total yield at the end of the rotation, the knowledge of growth dynamics is essential to determine optimal harvest cycles and to assess the influence of specific management choices or climatic conditions. Furthermore, the development of stand structures and the distribution of tree dimensions are also crucial for management decisions. For instance, for bioenergy purposes the maximum diameter is restricted by direct chip harvesting methods [28]. Also, the higher ash content in small diameter trees, which is mainly related to the amount of bark, lowers fuel quality [28, 29] and increases nutrient removal [24]. Besides, an unequal stand development may also lead to mortality of individuals, which may impact total biomass production during later rotations [30]. Competition is the main d river leading to changes in size and weight distributions within a stand and thus towards an increasing inequality between dominant and suppressed plants [31]. According to Tomé and Verwijst [30], competition between individual plants is enforced in SRFS, which mostly consist of one single species or clone where plants are genetically alike. Thus, they compete similarly for available resources. Furthermore, the spacing is dense and shoots are already under heavy competition during the first growing season. This is enhanced by the high initial growth rate of the species used in SRC and SRAFS, causing an earlier canopy closure and therewith an earlier onset of competition [30]. Although stand structure, growth dynamics, harvesting methods and wood usage are highly interdependent, studies about SRFS still often neglect stand hierarchies.

This study aims at closing the aforementioned knowledge gaps concerning growth dynamics and stand structure development of tree species under different growth-preconditions and to provide cultivation and usage recommendations. Therefore, both organic and conventional SRAFS of four tree species (black alder, black locust, poplar clone Max 3, willow clone Inger) were established in a long-term field trial at a research farm in southern Germany. The effects of species, age, and 21-year organic and conventional farming on yield and stand structure were evaluated by monitoring sprouting, stem diameter, tree height and aboveground woody biomass during the first four-year rotation of the SRAFS.

## 2 Materials and methods

### 2.1 Study area and agroforestry design

**Fig. 1:**
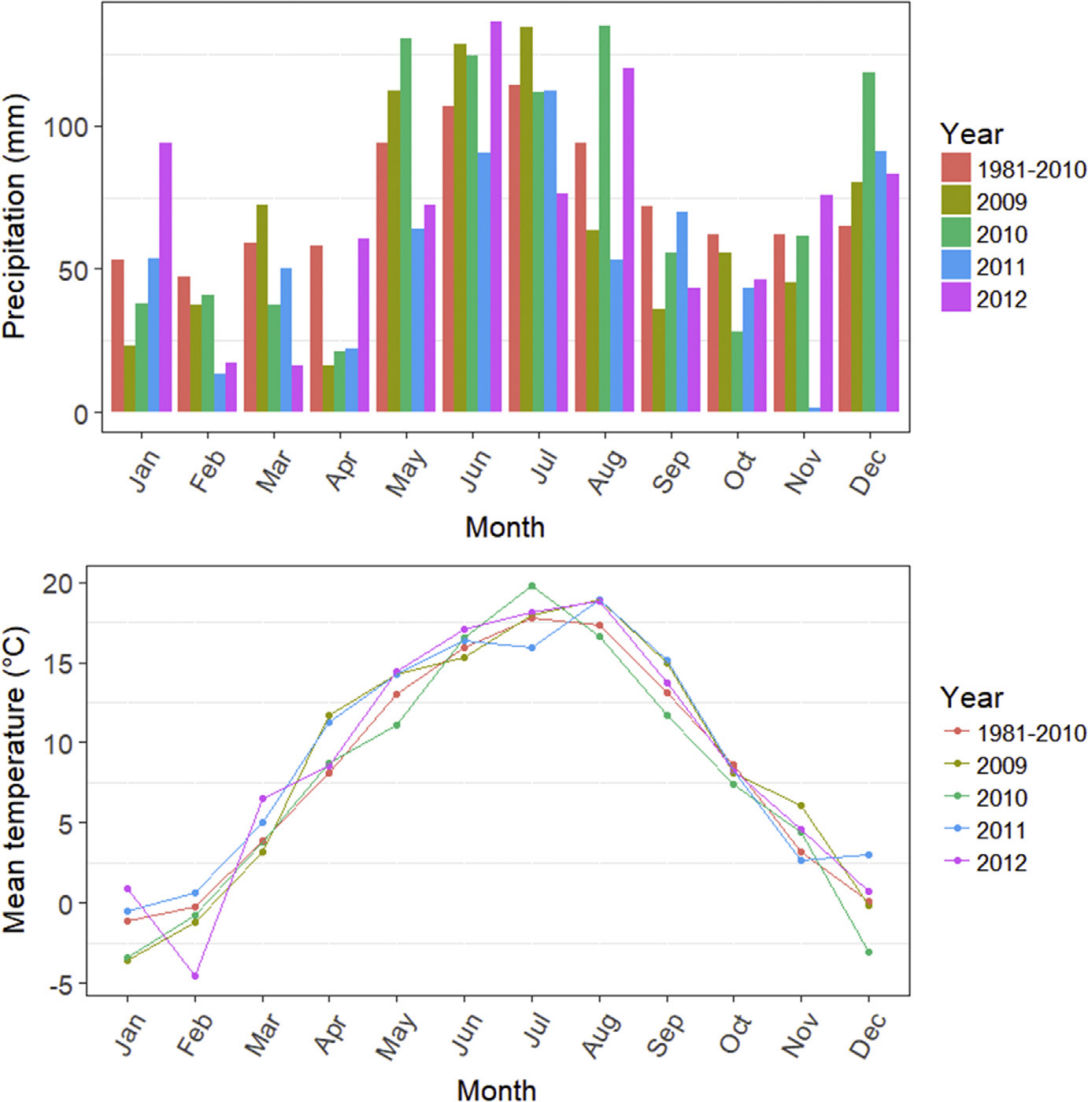
Sum of monthly precipitation (mm) and monthly mean temperature (°C). Data from a meteorological station near the experimental site showing both the long-term average for the years 1981–2010 and annual values during the first rotation from 2009–2012.

The study was performed at the Scheyern experimental farm (48°30′N, 11°21′E) in Bavaria, southern Germany. The farm consists of many independent fields in hilly terrain. Meteorological data were obtained from the nearby Altomünster-Maisbrunn weather station (48°24′ N, 11°19′ E) of the Deutscher Wetterdienst (DWD). The climate is temperate with an annual average temperature of 8.9 °C, 7.8 °C, 9.3 °C, and 9.0 °C and an annual precipitation of 804 mm, 902 mm, 664 mm, and 841 for the years 2009–2012, respectively (Fig. 1). The long-term average (1981–2010) is 8.3 °C and 887 mm. Precipitation during the growing season (April to September) of the establishment year 2009 was above average. The season 2011 was marked by a prolonged drought (April, May, June, and August).

In 1992, the farm had been subdivided into an organic and conventional farming system, and each system was applied to different fields. The organic system is a low-input system and since 2005 maintained as an organic arable farming without livestock. It is based on a seven-field crop rotation with 29% grass-clover-alfalfa, 29% winter wheat, 14% potato, 14% sunflower, 14% winter rye. Mineral nitrogen and chemico-synthetic plant protection products are omitted. Tillage is carried out with a moldboard plow. The conventional system is a high-input system with chemico-synthetic plant protection use, mineral nitrogen input (on average 179 kg N ha^−1^ y^−1^ for 2009–2012 [32]) and a simple structured crop rotation with 50% wheat, 25% forage maize, and 25% potato. Here conservation tillage is applied (no plowing, crop residue incorporation with a grubber, mustard catch crop). This systems has significantly higher agricultural crops yields [32, 33].

**Fig. 2:**
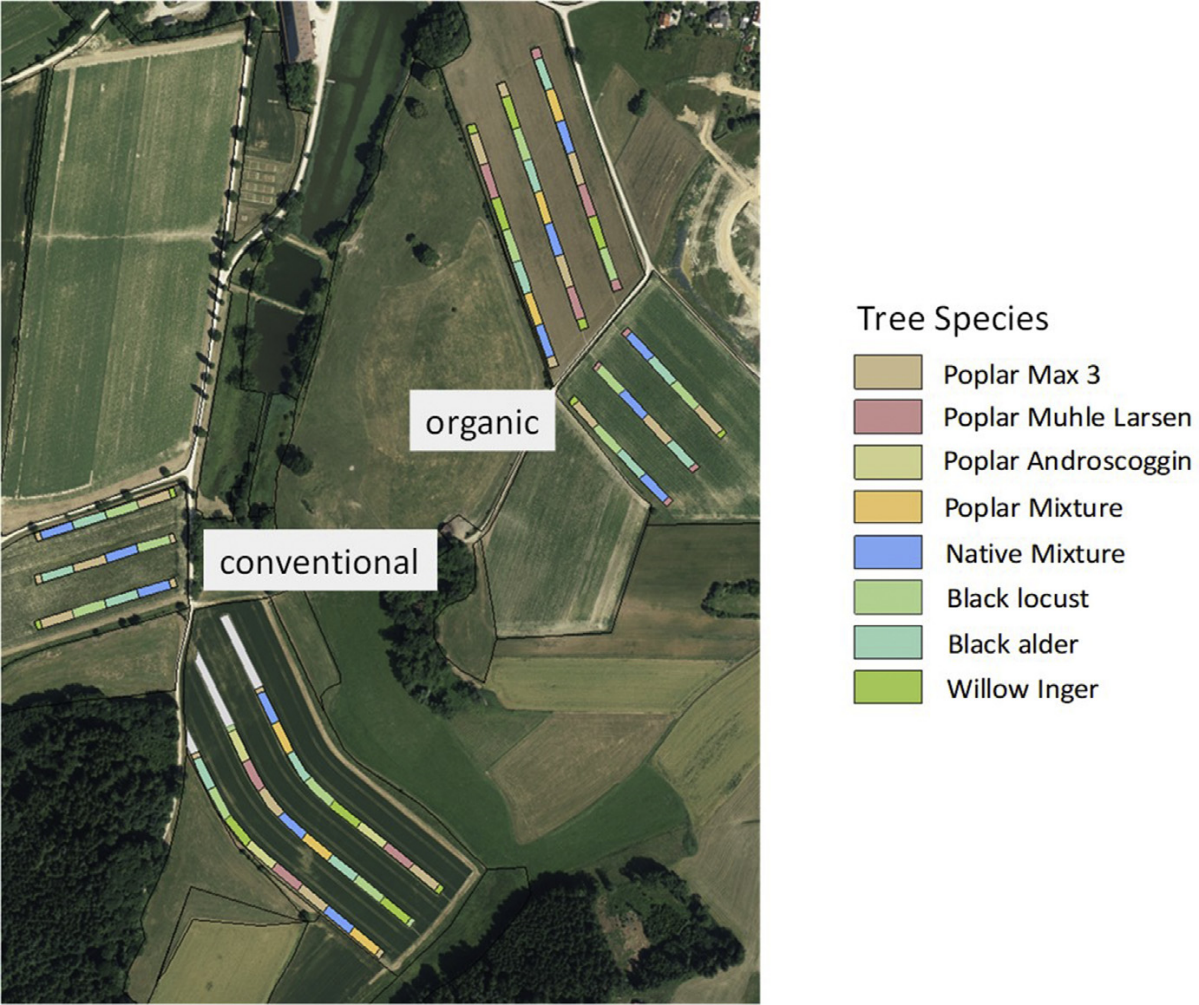
Experimental design of the agroforestry systems at the Scheyern experimental farm (48°30′N, 11°21′E) in Bavaria, southern Germany. Three strips of various species were planted on four fields (two organic, two conventional). Published in Hülsbergen et al. [34].

In April 2009, short-rotation agroforestry systems (SRAFS) were established in four fields of the farm, two for each farming system (Fig. 2). The altitude varies between 450 and 550 m above sea level with a 2–10% slope. Soils have a loamy texture and are classified as either Cambisols or Eutrochrepts with thin layer of loess, Cambisol with sand and gravel subsoil (sandy-gravelly illuvial horizon) or small-scale clay soils [35].

On every field, three strips of different fast-growing tree species were planted in a north–south (three of the four fields) or west–east direction (one of the four fields), with a spacing of 30 m for the field crops in between. Tree species were allocated randomly in blocks inside each strip. Inside each strip, trees were planted in three double rows (8.25 m wide) accommodating a density of 17,778 cuttings per ha. In this study, only the middle row was used for analysis because of significant border effects (see Huber et al. [36]). The studied species were black alder (*Alnus glutinosa*), black locust (*Robinia pseudoacacia*), poplar clone Max 3 (*Populus maximowiczii × P. nigra*), and willow clone Inger (*Salix triandra × S. viminalis*). Thus the study design is a randomized block design that includes 4 fields x 3 strips x 4 species. An exception is willow, which was only planted in two fields (one conventional and one organic). Such a design was chosen because it was impossible to randomize farming systems due to the agricultural constraints, and unfeasible not to plant the species in blocks. A more detailed description of the field experimental design has been published by Huber et al. [36].

Poplar and willow cuttings, approximately 20 cm in length, were planted manually to a depth of 15 cm. The other species, 70–90 cm in length, were planted manually as bare-rooted saplings. The tree strips were not manured, but weeds were controlled by herbicide application (conventional system) and mechanical weeding (organic system) in the first year of establishment. No further weed control or fertilizer applications were provided.

After more than 20 years, the farming system differences did not lead to a significant difference in the initial nutrient status of the soil of the SRAFS area (Table 1, published in Huber et al. [37]). Only soil organic carbon and potassium were slightly higher in the organic farming system, whereas phosphorus was slightly higher in the conventional farming system.

**Table 1:** Soil properties at 0–30 cm depth in conventional and organic farming systems at the beginning of the experiment in 2009.

Component	Unit	Conventional farming Mean ± SE	Organic farming Mean ± SE
C org	% by mass	1.11 ± 0.07^a^	1.17 ± 0.10^a^
N org	% by mass	0.11 ± 0.01^a^	0.11 ± 0.01^a^
pH		5.4 ± 0.1^a^	5.5 ± 0.2^a^
P	kg ha^−1^	5.2 ± 1.4^a^	3.5 ± 1.9^a^
K	kg ha^−1^	8.8 ± 1.4^a^	9.6 ± 2.0^a^

Mean organic carbon (C org), organic nitrogen (N org), pH, available phosphorus (P), available potassium (K), and the respective standard errors (SE).

Reprinted by permission from Springer Nature, Springer Nature, European Journal of Forest Research, Allometric tree biomass models of various species grown in short-rotation agroforestry systems, Julia A. Huber, Katharina May, Kurt-Jürgen Hülsbergen, © Springer-Verlag Berlin Heidelberg, 2016 (https://link.springer.com/journal/10342) [37].

a Farming systems sharing the same letter are not significantly different from each other (Tukey-HSD, p > 0.05).

### 2.2 Measurements

Measurements were made at the end of each growing season, on a selection of 10 individuals in the middle double row of each species in each strip (2.25 m *×* 2.5 m area), giving 120 individuals for each year and species (10 trees × 3 strips × 4 fields), respectively. Willow was planted on only two of four fields, resulting in one half of measured individuals, which is 60.

Stem base diameter (SBD, at 10 cm above soil) was measured for all shoots of an individual tree in two perpendicular directions using a caliper and the mean value was used in further calculations. Height (H; in m) was measured only for the dominant shoot using a Vertex hypsometer. Because most trees rarely developed more than two dominant shoots, sprouting performance was assessed by summing up the number of shoots of the measured trees and converting it to hectares. Dead trees were counted and replaced with other trees for measurements. Aboveground leafless dry biomass was estimated by allometric functions that predict individual tree dry biomass from SBD, retrieved from a previous study on the same study site [37],

(1) $$M_{i}=\beta_{0j}SBD_{i}^{\beta_{1}}$$

where M is the total aboveground oven dry mass for a specific stem base diameter SBD, *β*_*0*_ describes the allometric factor, and *β*_*1*_ describes the allometric exponent. The additional index *i* refers to the individual tree and index *j* indicates the species-specific factors listed in Table 2. The equation was applied to all shoots. Single-shoot biomasses were summed for each species plot and yield at stand level (in t ha^−1^) was calculated. Subsequently, mean annual increment (MAI) is calculated as the accumulated stand yield divided by stand age, while current annual increment (CAI) is the change in size in the current year. All biomass values are expressed as oven dry mass.

**Table 2:** Allometric coefficients to calculate aboveground biomass of different tree species, where β_0_ describes the allometric factor and β_1_ the allometric exponent with stem base diameter (SBD in cm) as explaining variable.

Tree species	*β*_*0*_	*β*_*1*_
Black alder	0.025	2.603
Black locust	0.041	2.603
Poplar Max 3	0.036	2.603
Willow Inger	0.037	2.603

Adapted by permission from Springer Nature: Springer Nature, European Journal of Forest Research, Allometric tree biomass models of various species grown in short-rotation agroforestry systems, Julia A. Huber, Katharina May, Kurt-Jürgen Hülsbergen, © Springer-Verlag Berlin Heidelberg, 2016 [37].

### 2.3 Analysis

Density curves were computed considering all or dominant shoots. For each distribution, skewness was determined to quantify size asymmetry by reflecting the proportion of large to small individuals [38]. The Gini-coefficient (G) was used to quantify size inequality [31], which is given by

(2) $$G=\frac{\sum_{i=1}^{n}\sum_{j=1}^{n}\left|x_{i}-x_{j}\right|}{2n^{2}\bar{x}}$$

where *x*_*i*_ and *x*_*j*_ are the sizes of individuals i and j, respectively. G reflects the variation between individuals or the dominance of the larger individuals, and when G equals zero, size equality is perfect.

While the study design includes three replicate strips inside each of the four fields, the four fields are so different to each other that the strips are not comparable between fields (except possibly the two organic fields, which are directly next to each other). Thus, a standard ANOVA was unfeasible. Instead, we chose to include a field-strip factor (4*3 = 12 levels) as a random effect in the analysis to account for any field-strip effects.

Mean biomass, SBD, H, and shoot density were each modelled separately using a mixed effects model. Only the dominant shoot was used to avoid underestimation. Each model included the three-way interaction between species (4 levels), farming system (2 levels, organic and conventional), and year (4 levels, 2009–2012). We explicitly chose to include year as a factor, since this study focuses on growth dynamics. Since the variation of the response variables (biomass, SBD, …) increased with year, model residuals were not homoscedastic and the residual variance increased with year. To account for this, a weighting of observations was introduced such that the error variance was allowed to vary by year, that is $Var\left(\varepsilon_{i}\right)=\sigma^{2}\phi_{year\left(i\right)}$, where $\varepsilon_{i}$ is the model residual of observation *i*, σ is the residual variance, *year(i)* is the year of observation *i*, and $\phi_{year}$ are estimated variance ratios for the years 2010, 2011, and 2012, relative to the first year 2009 with $\phi_{2009}=1$.

We are aware that the study design can only determine observationally any farming system effects. To test, whether we have not mistakenly interpreted farming effects as field effects, we conducted the same analysis as before but exchanged the Management factor with a field factor. Also, willow was dropped from this analysis since it was only planted on two fields. The results showed significant field-species interactions and that significant differences between fields occurred mostly between cropping systems and not within. So we left the Management factor inside the original model formulation, since for practioners it is of more value to know about management differences than field differences.

All computations and statistical analyses were performed with R software version 3.4.3. Models were estimated using the lme-function in R package nlme [39]. Pairwise differences between species and clones within each year and farming system were determined by a post hoc analysis (similar to Tukey's HSD for normal ANOVAs) using R package lsmeans [40]. P-values of the posthoc tests were adjusted for multiple testing. If not stated otherwise, p-values below 0.05 denote significance.

## 3 Results

### 3.1 Sprouting and mortality

**Fig. 3:**
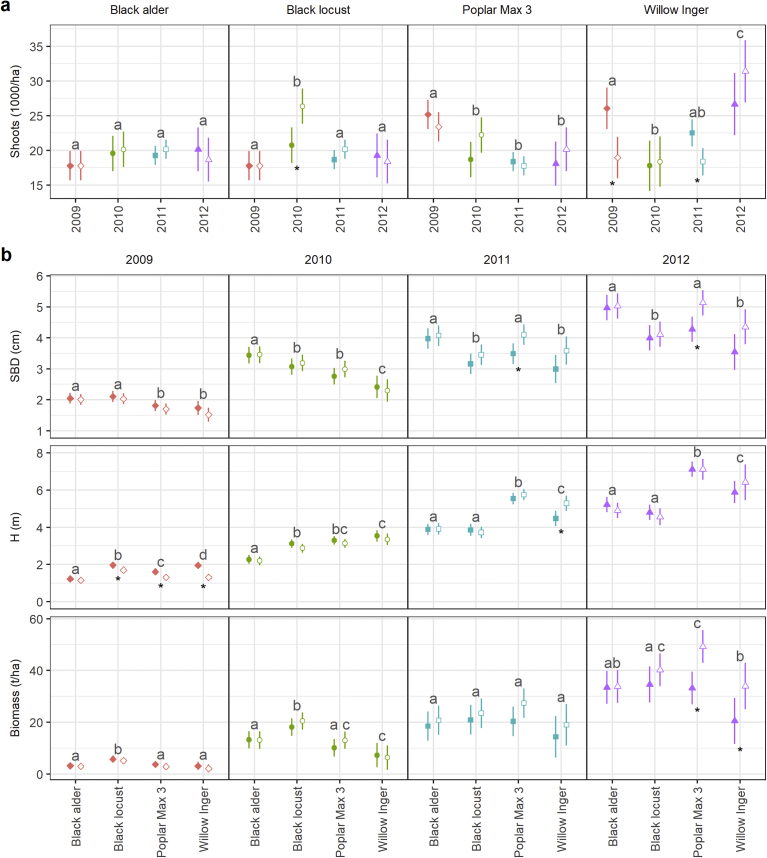
Modelled mean values of a) shoot density and b) stem base diameter (SBD), tree height (H), and biomass over the first rotation of the short rotation agroforestry systems. Shown are means with 95% confidence intervals of the conventional (solid points) and organic (hollow points) farming systems. In a) years were compared within species, and years sharing the same letter are not significantly different (p > 0.05). In b) species were compared within years, and species sharing the same letter are not significantly different (p > 0.05). Asterisks indicate significant differences between farming systems (p < 0.05). Biomass values were modelled by allometric functions using stem base diameter as predictive variable. To avoid underestimation, only the main stem was used for analysis.

Initial plant density was equal for all species (17,778), but sprouting and mortality resulted in different shoot densities during the rotation (Fig. 3) The mixed models revealed for the shoot density significant three-way interactions between year, species and management for (p = 0.049) (Table 3). After the first year, and more distinct at the conventional system, poplar and willow developed more shoots per tree, whereas black alder and black locust mainly had one shoot. In the second year, and more intense on the organic system (significant system difference for locust), sprouting was stimulated for black alder and black locust. 3% tree mortality was detected for conventional locust, 1% for organic alder. For poplar and willow no tree mortality was recorded and shoot density decreased, mainly at the conventional system (significant system difference for poplar). After the third year, shoot density had again increased for willow at the conventional system, but decreased for poplar and black locust mainly on organic systems. Stand density stayed on the same level for alder and organic willow. No further tree mortality was recorded in this year. While the shoot density had decreased after the fourth year for organic alder (9% tree mortality in total), organic black locust (3% tree mortality), and conventional poplar (4% tree mortality), it had increased for conventional alder (5% tree mortality), conventional black locust (13% tree mortality), organic poplar (no tree mortality), and willow on both systems (no tree mortality).

**Table 3:** ANOVA results for the mixed effects models of Shoots, SBD, Height, and Biomass. Shown are F and associated p values for a sequential ANOVA. Colon (:) in the variable column denotes interactions; df is degrees of freedom. See also methods section for full details on the mixed models.

Variable	df	Shoots		SBD		Height		Biomass	
		F	p	F	p	F	p	F	p
(Intercept)	1	13347.391	<.0001	3307.615	<.0001	2687.2729	<.0001	464.4279	<.0001
Vegetation period	3	4.766	0.0035	761.920	<.0001	2163.0368	<.0001	452.3879	<.0001
Tree species	3	5.237	0.0019	40.263	<.0001	114.7499	<.0001	24.2009	<.0001
Management	1	0.105	0.7531	0.064	0.8055	6.5943	0.0280	0.1020	0.7560
Vegetation period: Tree species	9	13.702	<.0001	9.398	<.0001	54.1796	<.0001	5.3453	<.0001
Vegetation period: Management	3	5.135	0.0022	9.140	<.0001	6.2916	0.0003	7.7991	0.0001
Tree species: Management	3	6.158	0.0006	0.582	0.6272	3.7447	0.0107	0.0925	0.9641
Vegetation period: Tree species: Management	9	1.963	0.0490	1.559	0.1224	3.0888	0.0011	1.3385	0.2238

### 3.2 Change of size distributions

**Fig. 4:**
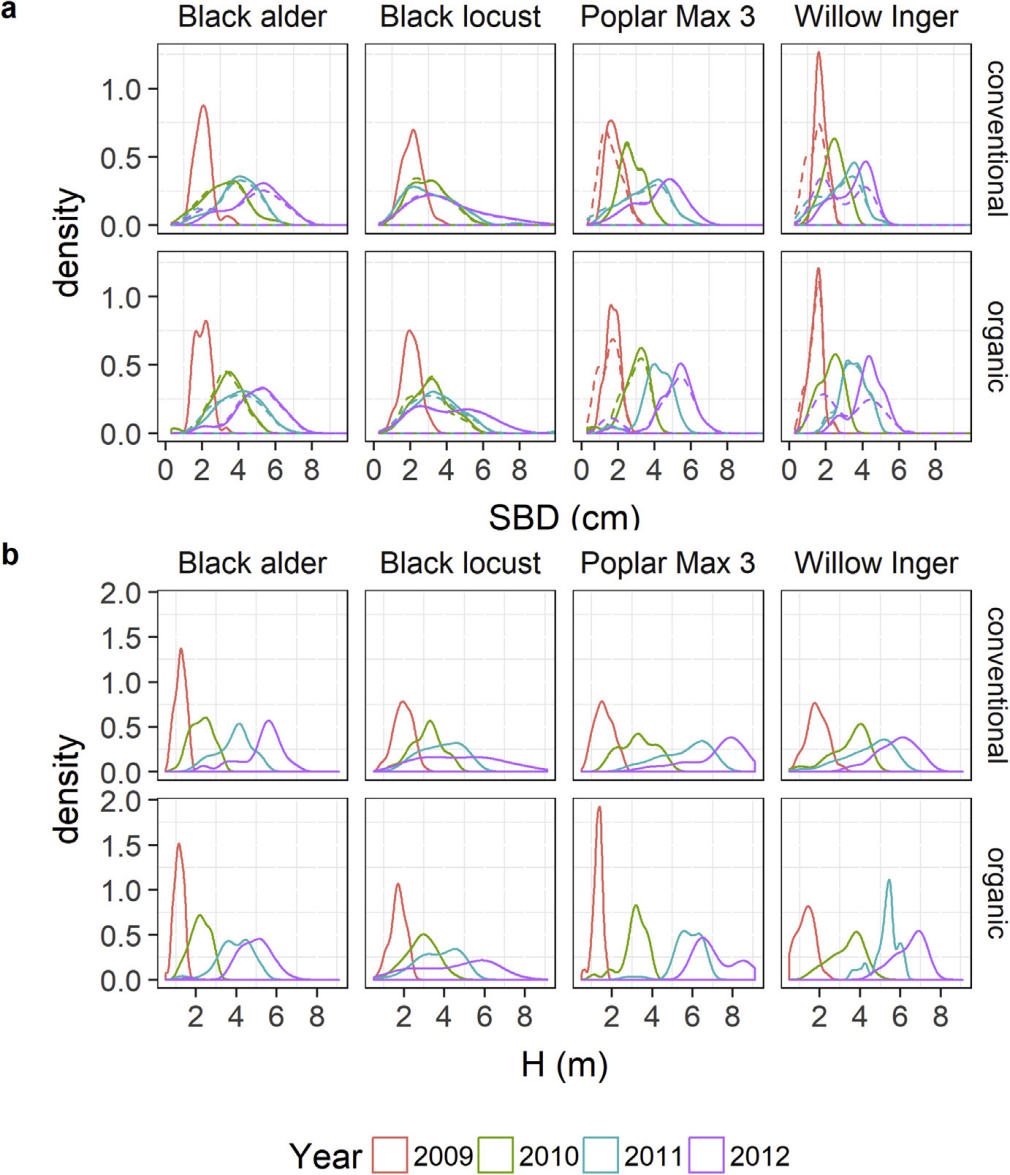
Density plots per year of a) the stem base diameter (SBD) and b) tree height (H) distributions of all shoots (dashed line, only for SBD), and of only main stems (solid line) in a conventional and organic short-rotation agroforestry system for different tree species.

**Fig. 5:**
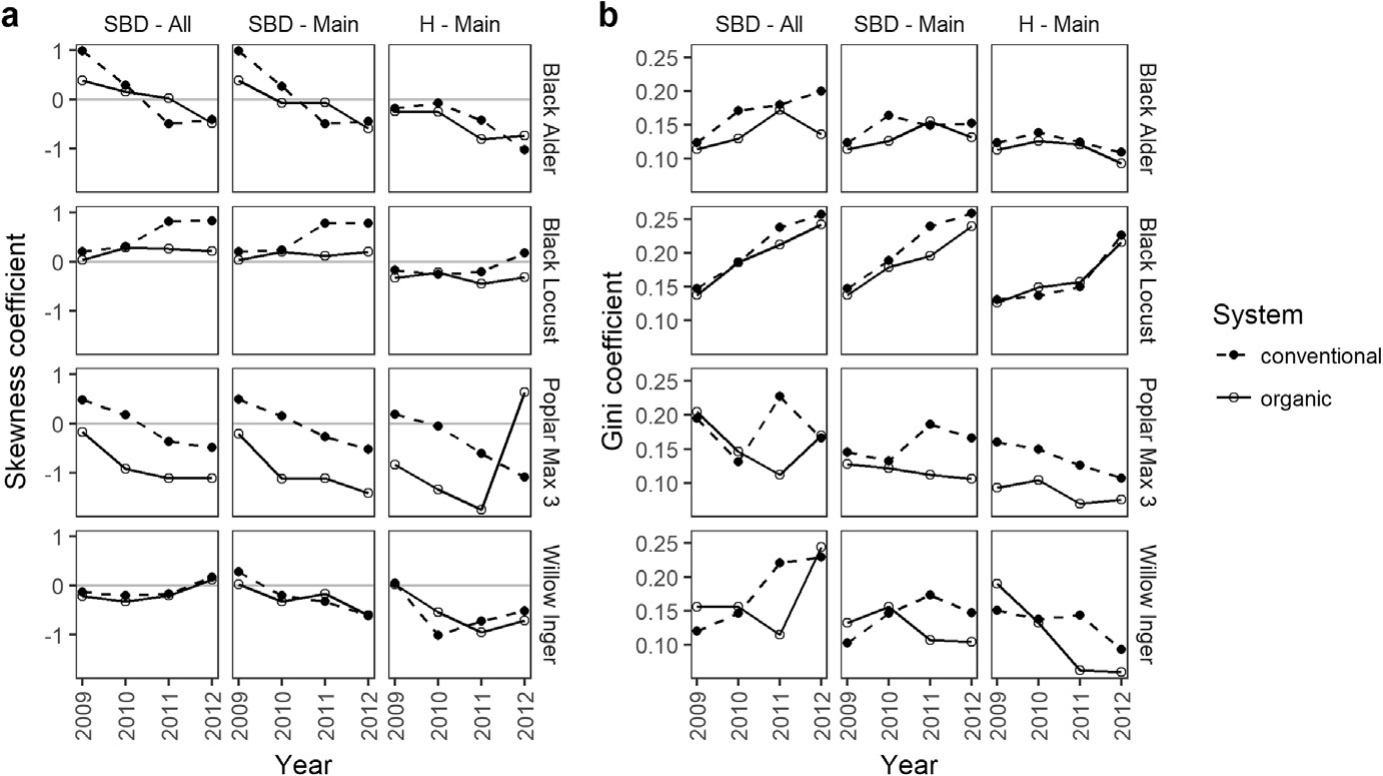
a) Skewness and b) inequality (Gini coefficient) of stem base diameter (SBD) and tree height (H) distribution of different tree species and farming systems after each year of growth. All shoots (All) or main stems (Main) were considered.

After the first year, all species except organic poplar stands, showed a positively skewed SBD distribution of the main stems (Figs. 4 and 5). During growth, the SBD distribution of black alder, poplar and willow became more and more left skewed. Negative skewness resulted from the presence of some very small individuals in the stands, with most values concentrated in the higher size classes (on the right side of the mean). The SBD distribution of locust stands were always right skewed and skewness increased during growth. Distribution of organic locust stands were less skewed and became almost bimodal in 2012, as those stands had a greater portion of big trees growing. H distributions started mostly left skewed (except conventional willow and poplar stands) and skewness mainly became stronger over time. Only organic poplar stands developed bimodality in the last year, and black locust distribution stayed more or less with the same skewness value.

Black locust developed the most unequal SBD and H distributions among all species. Inequality increased during the rotation due to growth of dominant trees and growth reduction of suppressed trees. For conventional alder, poplar and willow stands, the inequality of the SBD distribution was highest in the last two (poplar, willow) or three (alder) years, since a greater portion of small trees that were still alive stayed behind. Trees of the organic stands grew more equally and inequality decreased in the last (alder) or last two (poplar, willow) years. In the case of alder the mortality of small trees may have influenced this (Fig. 3). Inequality of the H distribution was lower than that of the SBD distribution due to a more equal development of the whole stand.

The inclusion of sprouts increased the amount of small trees and total size ranges and therewith inequality. In the last year, intensive sprouting at willow and organic poplar stands led to bimodality of their distribution.

### 3.3 Mean growth

The mixed models revealed significant three-way interactions between year, species and management for Height (p = 0.001), but not for SBD (p = 0.12) and biomass (p = 0.22); see also Table 3. For SBD and biomass, significant two-way interactions between year and species as well as between year and management were observed (all p < 0.001), while there were no significant interactions between species and management (both p > 0.05).

After the first year, mean SBD of alder and black locust was significantly higher than of poplar and willow (Fig. 3). Since alder and black locust were planted as bare root saplings, they had a substantial advantage over the other two species, which were planted as cuttings and therewith needed time to establish rooting systems. Additionally, mean H was significantly greatest for black locust, but significantly lowest for alder, whereas poplar and willow were in between. Consequently, black locust developed the significantly highest biomass (5.5 t ha^−1^), followed by poplar (3.4 t ha^−1^), alder (3.1 t ha^−1^), and willow (2.7 t ha^−1^). The conventional system had higher values than the organic, however, only significant for H of black locust, poplar and willow.

After the second year, the high current annual increment (CAI) of SBD for alder resulted in the significantly highest SBD among species, and the low CAI for willow in the significantly lowest SBD. Regarding H, poplar and willow had a higher CAI than the other two species. Therefore, the clonal ranking for H has changed: Willow had the greatest and poplar the second greatest tree height. Black locust had the highest CAI of biomass (13.9 t ha^−1^ a^−1^), followed by alder (10.1 t ha^−1^ a^−1^), poplar (8.3 t ha^−1^ a^−1^), and willow (4.1 t ha^−1^ a^−1^). For all tree variables, the organic system showed a higher mean CAI than the conventional. Subsequently, the differences in mean H between the systems had become lower. Furthermore, mean SBD and biomass of organic tree stands was even higher than of conventional stands, except for willow. This was also due to some small trees in the conventional system, reducing the overall mean.

In the third year, mean increase in SBD had lowered for all species, especially for black locust, which almost did not grow at all. However, the H increased and for alder and poplar even more than in the previous year. The CAI of biomass was reduced to 6.5 t ha^−1^ a^−1^ for alder and to 2.9 t ha^−1^ a^−1^ for black locust, but enhanced to 12.3 t ha^−1^ a^−1^ for poplar and to 9.9 t ha^−1^ a^−1^ for willow. Species ranking in terms of SBD had changed to black locust and willow having the significantly lowest values, and in terms of H and biomass to poplar having the highest values (only for H significant). Growth of alder and black locust was probably inhibited because of the low precipitation during the vegetation period 2011 (Fig. 1). Furthermore, a late budburst was detected for locust because of frost events in spring. Again, the organic stands showed higher CAIs for all variables compared to the conventional stands, except for H of locust. The difference was significant for SBD of poplar and for H of willow. Main reasons for this may be the larger stand separation in conventional poplar and willow stands (the reduced growth of the smallest) and the uniform growth of the whole organic stands.

After the fourth year, the tree species showed an elevated growth in mean SBD and biomass, but lowered growth of H, except for black locust. At the end of the rotation, poplar developed a mean SBD comparable to alder, whereas black locust and willow achieved significantly lower mean SBD. Mean height was significantly greatest for poplar, lower for willow and significantly lowest for alder and black locust. For all species the CAI of biomass was greatest in this year and with this also MAI, except for locust due to the low growth in the third year. The highest rotation MAI of biomass was observed for poplar (10.3 t ha^−1^), followed by black locust (9.4 t ha^−1^), alder (8.4 t ha^−1^) and willow (6.8 t ha^−1^) in decreasing order. The ranking of the clones in terms of SBD was not in agreement with the observed biomass production, since wood density, branching patterns and size distribution also play an important role. By summing up the single tree biomasses, which were greatest in black locust, high stand biomass can be achieved. Although mean diameter and height were lowest for black locust, the biomass production was still comparable to that of poplar. The organic stands of alder and black locust had lower CAIs of SBD and H, and for alder also of biomass, than the conventional stands. In contrast, organic poplar and willow had much higher mean CAIs than the conventional stands, strengthened by the suppression of lower diameter classes. Finally, mean SBDs were higher in organic stands, however only for poplar significant. System differences in terms of biomass were significant for poplar and willow.

## 4 Discussion

### 4.1 Development of size hierarchies

In the initial development stage of a stand, the rate of individual tree growth is solely a function of age, species and abiotic site factors. Small trees grow without competitive interaction because the site is not fully occupied and resources are not fully exploited [41]. Therefore, population recruitment may continue and subordinate weeds may establish [41]. Also at this study site, additional shoots emerged in the first or second growth period because of to the availability of space. The size distribution was mostly normal, which is typical before competition initiates [41].

When stands develop further, the growth of small trees is inhibited because of shading by taller neighbors, while larger trees are less affected and continue their growth [41]. Consequently, the size variability increases and the size distribution become increasingly skewed and unequal due to intraspecific competition. In our study, all species showed a strong increase of their size range as well as an increase of inequality and asymmetry with plantation age. Except for locust, the size distribution became negatively skewed due to the steady growth of the larger trees and suppression of some small individuals under the main canopy. Locust had the most unequal stand and in contrast to the other species a positively skewed distribution with only few dominant and many suppressed individuals. In the study of Laureysens et al. [38], poplar size distributions were mainly right skewed for most clones during the 4 year rotation, and skewness decreased markedly because of the rapid elimination of the smaller shoots. However, size distributions of clones with a slow mortality of the smaller shoots still remained highly skewed at the end of the rotation.

With certain increase of tree size, suppressed trees [41, 42] as well as shoots within one tree [43] die. This natural self-thinning coincides with canopy closure [41]. The intraspecific competitive ability of trees is species- and even clone-specific and determined by specific biomass accumulation strategies, tree dimensions, leaf morphologies and canopy architectures [42, 44]. In this study, locust, alder and poplar reached canopy closure. Subsequently, those stands not only developed a size hierarchy but also underwent a loss of shoots and whole trees during the rotation. With the absence of disease or disturbances this can be attributed to self-thinning. Tree mortality was highest in black locust and alder stands, which developed the most widespread crown, and has also been reported in Huber et al. [36]. The high stand inequality, the positively skewed distribution, and the higher tree mortality indicates a stronger decline of competitiveness for black locust than for all other species. Poplar developed a orthotropic monopodial trunk with narrow crowns and small branches [36, 37, 45], probably explaining a lower between-plant competition. In contrast to the other species, canopy closure of willow had not yet been attained since the trees mostly had not more than two thin and seldomly branched shoots (see also Huber et al. [36]). Therewith, no loss of trees was recorded, and even more, new sprouts emerged already in the third growth period leading to a bimodal distribution. Willows high ability to produce more shoots when space is available was already recognized [43, 46]. In the study of Cienciala and Lindroth [46], already during the second year of a coppiced willow plantation the mortality of the smallest individuals made the initial bimodality disappear and the weight-frequency distributions positively skewed. With longer rotations and progressing stand development, size distributions of willow, but also of poplar and alder, may have changed to being positively skewed as well. Competition for light may not be the main driving force for shoot elimination. The suppression and removal of smaller shoots may also be caused by limited resource supply from the roots, leader shoots restoring apical dominance [38], or climatic factors and pathogens [30].

### 4.2 Temporal evolution of biometric variables and biomass

The biomass of black locust was comparable to poplar, although its mean diameter and height were lower. This was because black locust had some high diameter trees and the highest wood density (0.60 g cm^−3^ [47]) compared to the other species (ρ_Alder_ = 0.40 g cm^−3^, ρ_Poplar_ = 0.34 g cm^−3^, and ρ_Willow_ = 0.34 g cm^−3^; [47, 48]). Species ranking in biomass changed in the third year because of the high growth of poplar and reduced growth of black locust, which was likely a result of the observed late budburst because of frost damage. Although physiological adaptation to cold climates was found for black locust, stem dieback and a lowered growth rate had also been reported in response to cold [49]. Black alder is reported to be relatively tolerant to late autumnal and early spring frosts [9], while frost tolerances of willow and poplar depends on clone [50].

In the third year, H growth rate remained high, whereas an overall growth decline of SBD was observed. This may be caused by the low precipitation in that year, which could have resulted in a soil water deficit. However, water limitation was shown to reduce H growth in favor of SBD growth, reducing the length of the hydraulic transport system and embolism risk [51]. In contrast, competition for light makes it advantageous to increase height growth relative to diameter growth [52]. In our study, tree height variability was lower than SBD variability, i.e. trees of different diameters had comparable heights. This underlines that subdominant trees enhanced their height growth at cost of their diameter growth to improve access to light [53]. This was particularly present for willow, poplar and alder, whereas locust showed a wider H range. Maybe a trade-off between resource allocation in H and SBD favored H growth despite the reduced water availability.

All tree species showed the highest growth rate in the fourth year. Also in the study of Heinsoo et al. [50] most willow trees performed best at the end of the first four-year rotation period, and Kauter et al. [28] recommend a minimum rotation length of 5–10 years for poplar species. For all species, the maximum biomass growth was probably not yet reached. This is mostly evident for willow that did not yet fully occupy the available space. Early coppicing of willow can promote multiple-stem regrowth, which is supposed to increase final biomass production [14, 18, 54]. Extending rotation cycles would also enhance productivity of the plantation. However, technical restrictions by tree diameter (black locust already developed SBDs up to 9.3 cm) according to harvest or wood usage [26] may put a limit to the extension of the rotation period.

Furthermore, after cutting the trees at the end of the rotation, resprouting can differ among tree species and throughout the next rotations. Therewith, biomass production may differ in the following rotations, possibly resulting in a change in species ranking. Here, further research on the following rotations is needed.

### 4.3 Influence of farming system and site differences

Nitrogen fertilization has been shown to increase woody biomass yields or early culmination of biomass increment [7, 55], even for black locust, in spite of its capability to fix atmospheric nitrogen [7]. Likewise, favorable edaphic conditions can also increase the biomass yields of SRC [18]. At our research station, the yields of the conventionally managed and fertilized crops exceeded those of the organically managed ones (for potato by 35% and winter wheat by 52% during 2009–2012; [32]). In contrast, the tree strips were not fertilized in both systems. Furthermore, weed control has been applied in both systems, thus minimizing the influence of weed competition. The nutritional status was relatively similar between the systems, although the previous long-term cultivation differed. However, in the previous study of Huber et al. [36], a positive effect of fertilizer that has been applied at the adjacent fields of the conventional system was detected on tree growth of poplar and willow border rows of the AFS. In the first year, conventional systems showed higher biometric values and biomasses than organic ones. In the beginning, mineral nitrogen may have promoted tree growth and emergence of sprouts, whereas in the following years the fertilizer may have been absorbed only by the border rows and therewith inner rows reacted differently.

Generally, large between- and within-field variations due to variable soil properties and micro-climatic differences (100 m altitude difference, 2–10% slopes) made it difficult to distinguish between management and site effects, which was stated as main reason for the system differences in the previous study on the same site but only on the last year [36]. Also other authors emphasized that short rotation woody biomass yields were highly variable and site-dependent with no response to fertilizer [56], or site specific reactions [57]. Black locust and alder showed an overall growth reduction in the third year due to frost for black locust and maybe water limitation for alder. Still, black locust and alder responded much lower to farming system and showed lower plot variability than poplar and willow, which may be because of the ability to cover the use of nitrogen from their symbiotic fixation. This highlights that tree species respond differently to changing environmental conditions determining their productive potential.

## 5 Conclusions

The chosen poplar clone was well adapted to the conditions of our study site displaying, high growth and low size asymmetric competition throughout the rotation. Willow also showed a high tree height growth rate, but low diameter growth and low wood density led to a low yield. Due to the lack of crown closure, sprouting was still stimulated in the fourth year. Coppicing willow after the first year might stimulate early growth of multiple stems, possibly leading to a better use of light availability and thus a higher total yield. Further research is needed here.

Black locust had a promising growth until late frost in spring caused a severe reduction in its productivity. Nevertheless, after four years its biomass production was the second highest among species. Black locust showed huge size difference and mortality within its stands. A lower planting density might reduce mortality and would save planting costs. However, this is subject of further investigation. Furthermore, the large diameters within black locust stands might be problematic when harvesting with a mowing cutter, what limits rotation lengths.

Alder showed a moderate growth among species, but was within expected yields. Alder developed like black locust an unequal stand possibly impairing the harvest and quality of the wood. High variability due to locational variation was present for willow and polar. Black locust and alder were less sensitive to their location. Furthermore, for the latter species no difference in growth traits between organic and conventional systems were found during the rotation, except greater height of conventional black locust in the first year. In contrast, poplar and willow showed significant higher values for organic farming after 4 years. However, it is unclear if this can be attributed to management or site effects. Thus, organic farming did not depress the productivity of the trees, offering high potential for short rotation biomass production under this system. Because of these gene-environment interactions, species performances may differ at other locations and management regimes, which include initial planting density in combination with rotation length, fertilizer, and irrigation. Furthermore, growth may be altered in the next rotations attributed to for example variations in shoot emissions, survival rate, and weather conditions.

## Declarations

### Author contribution statement

Julie Huber: Conceived and designed the experiments; Performed the experiments; Analyzed and interpreted the data; Contributed reagents, materials, analysis tools or data; Wrote the paper.

Michael Matiu: Analyzed and interpreted the data; Contributed reagents, materials, analysis tools or data; Wrote the paper.

Kurt-Jürgen Hülsbergen: Conceived and designed the experiments; Wrote the paper.

### Funding statement

This work is part of the research project ELKE (Development of an extensive land-use strategy for the production of renewable resources as compensation measures of the impact regulation in Germany), funded by the German Federal Ministry of Food and Agriculture and the Agency for Renewable Resources.

### Competing interest statement

The authors declare no conflict of interest.

### Additional information

No additional information is available for this paper.
